# Cross-National User Priorities for Housing Provision and Accessibility — Findings from the European innovAge Project

**DOI:** 10.3390/ijerph120302670

**Published:** 2015-03-02

**Authors:** Maria Haak, Björn Slaug, Frank Oswald, Steven M. Schmidt, Joseph M. Rimland, Signe Tomsone, Thomas Ladö, Torbjörn Svensson, Susanne Iwarsson

**Affiliations:** 1 Department of Health Sciences, Faculty of Medicine, Lund University, Box 157, 221 00 Lund, Sweden; E-Mails: bjorn.slaug@med.lu.se (B.S.); steven.schmidt@med.lu.se (S.M.S.); signe.tomsone@med.lu.se (S.T.); thomas.lado@med.lu.se (T.L.); torbjorn.svensson@med.lu.se (T.S.); susanne.iwarsson@med.lu.se (S.I.); 2 Interdisciplinary Ageing Research, Goethe University Frankfurt/Main, Frankfurt 60323, Germany; E-Mail: oswald@em.uni-frankfurt.de; 3 Italian National Research Center on Ageing (INRCA), Via Santa Margherita, 5-60124 Ancona, Italy; E-Mail: j.rimland@inrca.it; 4 Department of Rehabilitation, Riga Stradins University, Dzirciema iela 16, LV 1007, Latvia

**Keywords:** ageing, environment, innovation, housing, accessibility, functional capacity

## Abstract

To develop an innovative information and communication technology (ICT) tool intended to help older people in their search for optimal housing solutions, a first step in the development process is to gain knowledge from the intended users. Thus the aim of this study was to deepen the knowledge about needs and expectations about housing options as expressed and prioritized by older people, people ageing with disabilities and professionals. A participatory design focus was adopted; 26 people with a range of functional limitations representing the user perspective and 15 professionals with a variety of backgrounds, participated in research circles that were conducted in four European countries. An additional 20 experts were invited as guests to the different research circle meetings. Three themes illustrating cross-national user priorities for housing provision and accessibility were identified: “Information barrier: accessible housing”, “Information barrier: housing adaptation benefits”, and “Cost barrier: housing adaptations”. In conclusion, early user involvement and identification of cross-national differences in priorities and housing options will strengthen the development of a user-friendly ICT tool that can empower older people and people with disabilities to be more active consumers regarding housing provision.

## 1. Introduction

Social innovations are new ideas (products, services and models) that strive to address pressing current needs. As they simultaneously meet social needs (being more effectively than alternatives) and create new social relationships or collaborations [1], such innovations are social both in their ends and in their means. Facing the global challenges imposed by the rapidly ageing population, the need for social innovations that target issues related to the needs of senior citizens is immense, and the European innovAge project directly addresses this challenge by developing four social innovations to support active and healthy ageing (http://www.innovage.group.shef.ac.uk/).

According to the UN Department of Economics [2], by 2019, the population of people 65 years and older across the world will surpass the population of children aged 5 years and younger. More and more people are living longer periods of their lives with disabilities [3], which increases demands for accessible housing that supports active and healthy ageing. Although the legislation in many European countries places high demands for the design of accessible housing, insufficient accessibility is still a major problem [4]. For example, many people ageing with disabilities live in dwellings with stairs at the entrance, without elevators, and with many environmental barriers in the immediate outdoor surroundings [5]. Accessibility housing is a prerequisite for active and healthy ageing [6]. To accommodate the needs of the rapidly ageing population, systematic surveys of environmental barriers represent a strategy that can contribute to the identification of accessibility problems in the housing stock, which could then be targets for the development of innovative solutions. The present study, which is one out of four social innovations of innovAge, aims to develop a new information and communication technology (ICT) tool, based on research with the involvement of senior citizens from several European countries including those ageing with disabilities. The new ICT tool is intended to be used by older people to analyze person-environment (P-E) fit in home and neighborhood environments and to empower them by increasing their awareness about housing options and facilitating their active involvement in choosing accessible housing. That is, we envision that senior citizens will become more critical consumers and exert pressure on practices and policies regarding accessible housing and housing provision. With the present study we present the knowledge gained in the first step in the development of this product.

Research on housing and health among very old people in Europe has shown that home and neighborhood environments affect older people’s daily activities and participation, and adequately designed housing positively affects independence [7,8,9]. The home is the most important living space of older people. It is in their home environment most daily activities occur [10]. It is widely known that participation in daily activities gives meaning to people’s lives and participation is considered a determinant of well-being and health [11]. Older people themselves stress the importance of having a functional and secure home in order to be as independent and autonomous as possible in daily activities and maintain participation [7,12]. However, as people age, in general they often do not consider the health benefits of being more proactive in their choice of housing, and few individuals use proactive strategies in this respect [13]. Thus, there is a significant developmental potential to support older people to become more active consumers in the context of housing provision. In particular, those ageing with disabilities could benefit from well-informed choices about suitable housing when refurbishment or relocation is necessary. One can think of different types of solutions, but a solid methodology with the potential to foster user-driven housing for older people is called for.

The Housing Enabler (HE) is one well-established research-based methodology currently available for professional assessments of housing accessibility [14,15], and it has the potential to be modified for end user assessment of their own housing needs. It is useful not only for research and education but also to support practitioners in producing reliable and valid assessments as a basis for interventions targeting housing accessibility problems, at individual and group levels [16]. With the ecological model of ageing [17] as the theoretical base of the instrument, accessibility is operationalized as the relationship between the person’s functional capacity and the demands of the physical environment [18]. Accessibility is thus a relative concept comprised of a personal and an environmental component, and it is objective by nature as the environment is described on the basis of standards and guidelines for housing design [19]. The HE consists of a three-step assessment and analysis based on one checklist of functional capacity in the individual (personal component) and one checklist of environmental barriers (environmental component), followed by an analysis of P-E fit resulting in a quantitative measurement of accessibility problems [16]. Research using the HE has shown that environmental barriers are common in housing across Europe [20]. Moreover, when comparing very old people ageing with a chronic progressive disease such as Parkinson’s disease with very old people in general, it has been found that the number of environmental barriers does not differ. That is, given their more complex profiles of functional limitations, people with Parkinson’s disease are subjected to more accessibility problems, which implies the need for a more nuanced view on the housing situation for groups ageing with different disabilities [5].

At the prospect of the development of a new ICT tool with the potential to facilitate users to advocate for and meet their own needs, wishes and goals in terms of accessible housing, the aim of the present study was to deepen the knowledge about needs and expectations regarding housing options as expressed and prioritized by older people and people ageing with disabilities (*i.e.*, end-users) in different European countries.

## 2. Methods

A fundamental part of the development of an interactive and user-oriented ICT tool is the use of research methods that emphasize collaborative action between researchers and participants with different backgrounds but with a shared interest to influence and change a certain situation. To achieve this type of collaboration, we used the research circle methodology, which has its point of origin in the Swedish tradition of study circles [21]. It has been further developed by researchers at Lund University, Sweden to be a practice-oriented and structured research approach [21,22]. The theoretical foundation developed by Ferrière, Ferrer and Freinet goes back to the early twentieth century [21]. Throughout this paper the term research methodology is used when referring to the methodology itself; the term research circle relates to the actual group of individuals; and the term research circle sessions refers to the series of meetings that occurs in the context of the research process.

The research circle methodology is used as a means to engage participants representing the users, practitioners and researchers in a joint effort to discuss scientific knowledge in relation to reality and to develop or collect knowledge that has not previously been identified. The research circle methodology differs from the nearby focus group methodology in adopting an explicit participatory design focus where researchers and participants contribute with equal authority. That is, while focus groups are based on a group interview methodology, research circles represent a way to collaborate with users in the generation of knowledge.

Research circles include a selected number of people with different backgrounds who come together several times for a predefined period of time in workshop sessions to discuss a common topic. Applying the research circle method, we aspired to nurture communication among participants with different backgrounds with a common goal of generating ideas and suggestions about the topic under discussion, namely accessibility and housing provision for older people and people ageing with a disability. The HE and the person-environment fit model were used as the conceptual foundation throughout the research process. The research circle sessions provided a place for reflection in which the participants could describe and analyze problems and pose questions about housing provision, the built environment, environmental barriers generating accessibility problems, and the possible benefits of an ICT tool for the identification of optimal housing options.

### 2.1. Research Sites and Sampling Procedure

Four research circles were formed to compare housing in countries with quite different building traditions, legislation, policies for housing provision for senior citizens, and housing standards. That is, one set of research circle sessions was arranged in each of the following countries: Sweden, Latvia, Germany and Italy. In each nation, a research team was composed of at least one researcher and one research assistant. Guidelines were developed to describe the proper organization and implementation of the research circles and for the collection of data in a structured consistent manner. The guidelines were distributed to the participating research teams. Furthermore, for exchange during the process of successive data collections, regular Skype and personal meetings in workshops were arranged during the data collection period.

To promote creative discussions in the research circle meetings, purposeful sampling [23] was used in all four research sites to recruit participants with diverse backgrounds. The participants representing the end-user perspective were recruited from a variety of user organizations with the exception of Germany, where all participants were recruited from another research project. User organizations through which participants were invited were pensioners’ organizations and disability specific organizations. A mindset of readiness for change [24] was an inclusion criteria for participation, which means the potential participants had a positive attitude and were motivated for changes within housing provision. Further inclusion criteria were interest in accessibility issues, working with such issues or housing provision, and being committed to attend all four research circle meetings. An additional inclusion criterion was some experience using a computer. We sought a diversity of age, sex, civil status, functional capacity, use of mobility devices, types of housing lived in, and geographical districts. A sampling matrix was used during recruitment to ensure the achievement of this diversity. The participants representing the user perspective should be older people, but in Italy the participants recruited were somewhat younger due to the fact that actual organizations were invited to participate, rather than single individuals and each organization was free to select a representative to take part in the research circles. Thus, the age of these representatives could not be easily controlled by the researcher. Characteristics of the participants are shown in Table 1. The Internet was used to search for professionals within municipalities, private and public housing companies, architects working with accessibility issues or the like in order to identity professionals with valuable knowledge about accessibility and housing provision for older people and people with disabilities. Professionals and invited experts who took part in the research circle sessions in each country are described in Table 2. Professionals were participants in the research circles while experts were invited to single meetings.

**Table 1:** Characteristics of participants representing user perspective in the research circles (*n* = 26).

Characteristic	Sweden (*n* = 7)	Germany (*n* = 8)	Latvia (*n* = 6)	Italy (*n* = 5)	Total (*n* = 26)
*Sex n (%)*					
Men	4 (57)	4 (50)	3 (50)	3 (60)	14 (54)
Women	3 (43)	4 (50)	3 (50)	2 (40)	12 (46)
*Age group n (%)*					
<55	0 (0)	0 (0)	0 (0)	4 (80)	4 (15)
55–64	0 (0)	0 (0)	0 (0)	0 (0)	0 (0)
65–74	5 (71)	0 (0)	5 (83)	1 (20)	11 (42)
75+	2 (29)	8 (100)	1 (17)	0 (0)	11 (42)
*Disabilities/functional limitations n (%)*					
Visual impairment	1 (14)	1 (12)	1 (17)	2 (40)	5 (19)
Loss of hearing	1 (14)	3 (38)	0 (0)	2 (40)	6 (23)
Reduced mobility	1 (14)	5 (62)	4 (67)	2 (40)	12 (46)
Limitations in upper extremity	2 (29)	3 (38)	5 (83)	1 (20)	11 (42)
No functional limitations	4 (57)	2 (25)	1 (17)	0 (0)	7 (27)
*Use of mobility devices n (%)*					
Wheelchair	0 (0)	0 (0)	1 (17)	2 (40)	3 (12)
Wheeled walker	1 (14)	1 (16)	0 (0)	0 (0)	2 (8)
Cane	1 (14)	4 (50)	1 (17)	1 (20)	7 (27)
*Living situation n (%)*					
Living alone	1 (14)	6 (75)	1 (17)	3 (60) ^a^	11 (42)
Living with partner/married	6 (86)	2 (25)	5 (83)	1 (20) ^a^	14 (54)
*Dwelling (%)*					
Apartment	4 (57)	5 (63)	6 (100)	5 (100)	20 (77)
One-family house	3 (43)	3 (37)	0 (0)	0 (0)	6 (23)

Notes: **^a^** one participant lived with his parents and siblings; therefore he did not live alone nor with a partner. More than one functional limitation and more than one mobility device could be reported.

**Table 2:** Professionals in the research circles and experts invited to single meetings, *n* = 35.

Country	Professionals, *n* = 15	Invited Experts, *n* = 20
*Sweden*	Architect (1) Retired real-estate agent (1) Grant manager housing adaptation (1) Accessibility consultant (2)	Occupational therapist (2) Business developer (1), from a multinational construction and development company based in Sweden
*Germany*	None	Architect (2), one of them is a city planner Nurse (3), one is also a remedial teacher, one is an educational scientist at goethe-university frankfurt am main, and the third is also a phd student at Fachhochschule Frankfurt am Main Counselors (7), all working in social communities. The fields of work are: living counseling; living counseling and open senior services; ambulatory services; emergency call system; psychosocial counseling and support; counseling for independent lifestyle in old age; accessibility Student (1), age of 15 years (interested in technology)
*Italy*	Architect (3): one from a public housing estate company, 1 expert in city planning, 1 expert in design Urban planner (1) Regional office of research and innovation (1) Phd student architect (1)	Engineer (2), one expert in home adaptation and one expert in Smart Home Technology
*Latvia*	Architect/interior designer (2) Occupational therapist (1) Real estate project developer (1) for seniors	Doctor and IT, it-specialist and programmer (1), practicing in mobile application development Interior designer (1), practicing in housing development for seniors

All professionals and participants representing the user perspective were contacted by telephone. During this phone call, a research assistant assessed whether the potential participant fulfilled the inclusion criteria. The potential participants were informed about the scope and the goals of the project. Thereafter, letters with the same information as that given during the phone call were mailed. Next, repeated phone calls were initiated by a research assistant to schedule the dates for the set of research circle sessions. Finally, information on dates and locations for the research circle sessions were sent in a letter to each participant. Participants representing the user perspective also received a registration form with questions about basic participant characteristics which they completed and returned at the first research circle meeting.

In total, 22 out of 26 were 65 years or older. The age distribution was skewed to younger people in Italy (40% <55 years) and to older in Germany (100% 75 years or older). Both motor and sensory disabilities were represented among participants in all countries. Wheelchair users were represented in the research circles in two of the countries, and users of mobility devices were included in all four. There was an even distribution between men and women (55% and 45%, respectively).

In Germany there were no professionals among the regular participants of the research circle. Instead, there were several experts invited to each meeting representing the perspective of professionals. In Sweden, Italy and Latvia, architects were regular participants while in Germany one architect was invited as an expert. Municipality representatives such as housing adaptation or accessibility consultants and urban planners took part in the research circles in all countries. In addition, a wide variety of invited experts participated: nurses, doctors, engineers, IT specialists, occupational therapists, and construction company representatives.

### 2.2. The Procedure for Research Circle Meetings

**Figure 1:**
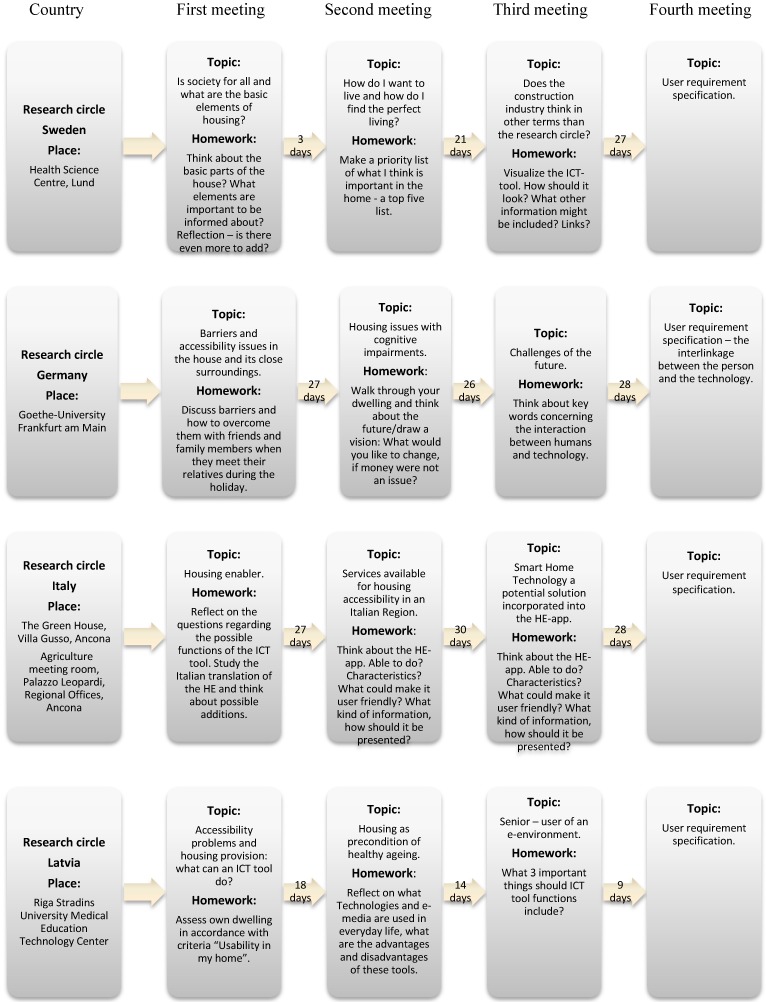
Topics discussed and homework assignments in each country decided by each research circle from meeting to meeting. In Italy it was the same homework assignment between meetings two and three.

Each research circle group met four times during a four-month period from March to June, 2013. Each meeting was led by one researcher and one research assistant and involved 7–14 participants with different backgrounds. All in all, a total of 41 people participated in the set of four research circle meetings (see Table 1 and Table 2). Each meeting lasted approximately three hours including breaks for refreshments. The research circle leaders strove to create an open supportive environment so all participants would feel safe and comfortable sharing ideas and information during the meetings. The research circle leaders spoke slowly and in a clear and accurate manner. They followed-up and encouraged reflections. Extra focus was put on making sure the participants felt that they contributed to the discussions and that they were not interrupted. When appropriate, in order to facilitate the discussion and generation of ideas, the research circles were divided into sub-groups during parts of the meetings. Efforts were also made to keep the discussion within the topic decided upon in advance. Themes for the first and the fourth meetings were determined in advance by the research circle leader (see Figure 1). The participants chose the themes for meetings 2 and 3, and they decided upon homework assignments for meetings 2 to 4. The rationale behind the use of homework assignments in between the research circle meetings was to nurture the richness of the discussions by stimulating the participants to be active, creative and motivated. The homework assignments encouraged the participants to discuss certain topics with other people in between the meetings and thus bring back additional information to discuss at the next meeting. Each group also chose experts to invite for the forthcoming meeting (see Table 2), and invitations to the suggested experts were then effectuated by the research circle leaders. The discussions at each research circle meeting produced the major part of the data used for the analysis.

Data were collected in the form of notes taken by the researchers during each meeting and by audio recordings. At various points in time, when the research circles were split into sub-groups, two or more digital recorders were used, and the researchers and the research assistants independently took notes in each group. The data collected during one meeting were then summarized and fed back to the participants at the next meeting. In this way, every meeting produced raw data and summaries. After each meeting, all summaries were translated into English and sent to the Swedish research team along with the sampling matrix, audio files, raw data and all other material used or produced.

### 2.3. Analysis Procedure

Data were analyzed separately for each country, and then used in a cross-national analysis. For the analysis of the research circles we adopted a pragmatic approach inspired by content analysis of manifest content [25], a method widely used within the qualitative research tradition. Content analysis is a systematic description of written, spoken or visual data, and according to Patton [23], a pragmatic scientific approach to analysis is relevant, especially in applied research.

The analysis procedure included two levels. Level one constituted an iterative process that began after the first meeting of the research circle [22] in each country. A summary of notes taken by the research circle leaders during the meetings formed the basis of an emerging categorization of data. Meetings 2 to 4 each started with a member check. That is, the participants were asked to provide feedback on whether the results of the previous meeting were properly reflected. In this way, the participants verified the accuracy of the analyses in connection to every step of the process (*i.e.*, every research circle meeting) and the participants became actively involved in the analyses. The main analysis at level one was done by the research circle leader in each country after each consecutive meeting. The research circle leaders gained insight into the data by reading the summary of notes together with the feedback from the participants. After the completion of each round of research circle sessions in all four countries, cross-national video link meetings were arranged. Led by the Swedish team, the research circle leaders in the countries involved monitored and gave feedback on issues raised for discussion by the partner countries. This approach strived to get further insight into the emerging bulk of data. Except from housing and ICT related issues the discussions concerned, for example, methodological questions regarding interview techniques or how to keep the discussions going within the target topic during each meeting. Level two of the analysis process involved only the research circle leaders in each country. At this level, insight into the data was gained by reading and discussing the content in all four research circle sessions. The data collected were gradually organized, and the researchers jointly analyzed how the different categories were related to each other. This procedure ensured the validity and credibility of the emerging categorization and was repeated in an iterative analysis process. Successively, the results of all 16 research circle meetings were synthesized.

### 2.4. Ethics

The ethical guidelines in each country were adhered to. According to current national legislation in the four countries, formal ethical approval was only needed for the Swedish arm of the project. Approval was granted by the regional Ethical Board in Lund, Sweden (2013/60).

## 3. Results

### 3.1. Process of the Research Circle Sessions

The topic for the first meeting in all countries was accessibility problems. For the second meeting, variations of housing for healthy ageing were discussed; Sweden and Latvia focused on the optimal housing, Germany focused on cognitive impairments, and Italy focused on services in relation to housing. For the third meeting, there was an emphasis on the future in terms of technical solutions in Germany, Italy and Latvia while in Sweden the builder’s point of view about accessible housing was discussed. The topic for the fourth meeting, in all countries, was to agree upon a specification requirement for an ICT tool. Regarding the homework assignments decided upon by the research circle participants from meeting to meeting, barriers were discussed in between two of the meetings in Sweden and Germany. In three out of four countries, homework assignments concerned reflections upon important aspects of the home environment. Following research circle meeting two or three, in all countries, there was a focus on technical solutions and interactions with the person and on what the ICT tool should be able to accomplish.

### 3.2. Cross-National Perspectives on Housing Provision and Accessibility Expressed by Users and Stakeholders

Based on the discussions in the set of research circle sessions across countries, three categories that illustrate the participants’ view on housing provision and accessibility issues were identified: “Information barrier: accessible housing”, “Information barrier: housing adaptation benefits”, and “Cost barrier: housing adaptation”.

#### 3.2.1. Information Barrier: Accessible Housing

In Sweden, Latvia and Germany, participants emphasized that seniors perceive it as difficult to find suitable information on accessible indoor, outdoor and neighborhood environments. In contrast, the Italian participants considered it relatively easy to search for information through the Internet. In addition, the participants in Sweden, Latvia and Germany viewed themselves as not well informed about existing laws and regulations regarding housing adaptation or whom to contact in order to receive information about accessible housing. All participants stressed the importance of having central or web-based platforms available to gather information, obtain advice and to consult, for example, regarding housing adaptations and refurbishment.

In Sweden and Germany, communication with different authorities was raised as an important issue. The participants perceived the answers from municipality professionals as sometimes being unclear. Furthermore, the participants stated that being frail made it harder to stand up for themselves, to raise their voices and to make requests for accessible housing when communicating with different authorities. For example, it is not easy for a new tenant with functional limitations to request home modifications from the landlord. Especially in Germany, it was perceived as problematic to ask the landlord to solve accessibility issues.

#### 3.2.2. Information Barrier: Housing Adaptation Benefit

In all four countries, lack of information about the risks of more or less accessible environments and their potential impact on everyday life was raised as a weakness. To raise awareness about one’s own environment and be able to request changes to make housing more accessible, more knowledge was sought about what impact different environmental barriers can have on individuals. For example, when moving to an unfamiliar environment, such as a new town, or when one’s health deteriorates, participants believed they did not have enough knowledge about the impact of the environment on their independence and participation. The general belief was that individuals do not become aware of environmental influences until something happens to them that affects them day-to-day. Such knowledge is useful not only for senior citizens, but also for professionals working within housing provision. This would help the latter to work preventively regarding accessibility issues. For example, the Latvian research circle members reported that a large number of apartment buildings built during the Soviet period and based on the standard of that time were associated with accessibility problems for individuals with functional limitations.

#### 3.2.3 Cost Barrier: Housing Adaptation

All participants, in various ways, raised the issue of high costs associated with refurbishment, housing adaptations, or rents in newly built accessible housing. In Sweden, where it is possible to apply for funding for housing adaptations if they have functional limitations, the focus was on the high rent for newly built accessible housing. Due to an already poor financial situation caused by disability, this makes it more difficult for people with disabilities to move into more suitable housing. Therefore, many people decide to remain where they are already living. While the participants of the Italian research circle stressed high costs for housing adaptations in general, the participants in the German research circle highlighted the costs associated with having to re-establish an adapted apartment when moving out. The research circle in Latvia raised the issue of not being able to do any modifications at home due to very limited private economic resources.

## 4. Discussion

The aim of this study was to identify critical issues associated with accessible housing and housing provision in order to develop an ICT tool. Applying an explicitly participatory, user-oriented approach that the research circle methodology allowed was a means to achieve that end. The HE and the person-environment fit model were used as the conceptual foundation throughout the research process.

The results revealed new knowledge and insights on common concerns and cross-national differences in how older people and people with disabilities express and prioritize their needs and expectations regarding housing options. Also, the explicit user perspective used to generate priorities and suggestions for technical solutions was fruitful. The knowledge generated by the present study will be used to provide user-driven input into the relevant content and functionality of a new ICT tool for accessible housing and housing provision for senior citizens.

A common concern expressed by the participants was that they were unable to take action to improve their housing accessibility due to lack of knowledge. They believed that with appropriate knowledge about potential environmental barriers in housing and neighborhoods, they would be able to proactively change their situation to prevent accessibility problems and subsequent activity limitations and participation restrictions. Another common concern expressed by the participants involved lack of knowledge and awareness of environmental impact on everyday life, and they considered this as a weakness. Subsequently, participants realized that early modifications to their existing housing environments when facing the development of functional limitations could prevent restrictions in everyday life or improve their ability to make well-informed decisions when considering relocation. This facet of our findings supports the advantage of having an ICT tool available in order to be make users aware of environmental barriers and accessibility problems. Such a tool could make it easier for people with functional limitations to continue living in their existing homes and/or help them find a more accessible home to move to. Even though concerns about accessibility trigger thoughts about housing adaptations or relocation [26], other aspects such as social and emotional issues play an important role in the decision making process [27]. Still, the need for information and raising of awareness should not be underestimated. According to Stans *et al.* [28], lack of power of clients negatively influences the communication between clients and professionals. Thus, in order to pave the way for a widespread use of an ICT tool with potential to support senior citizens to become more critical consumers regarding housing options, there is an immense need for active communication of knowledge about home and health dynamics along the process of ageing.

The present study also generated new insights into cross-national differences on housing for older people. While European policy-makers have been aware of economic differences between countries in Europe for many years [29], our project highlights some of the important economic challenges not only in eastern European countries such as Latvia but also in a wealthy country like Germany. For example, the unwillingness to ask a landlord to make home modifications due to fear of an increase in rent was a major concern in Germany, which apparently hampered measures that otherwise could have been taken to improve housing accessibility. In Sweden by contrast, this was not a concern as housing adaptations are covered through municipality-financed grants [30]. Such differences across Europe and how they impact possibilities for people to effectuate changes in their housing situation along with functional decline deserve more attention in research, practice, and policy. Even though housing policies are the responsibility of national governments, the European Union countries have a common goal of promoting economic and social integration [31]. In this context, housing policies play an increasingly important role because of the growing proportion of older people living in homes that are not adequately adapted to their needs.

As elucidated by the findings, the Italian research circle participants did not experience information or communication problems to the same extent as those of the other countries. One can reflect upon whether the ICT tool should be open for use through the Internet or if it should be framed as a personalized tool available only on a local hardware unit. Differences in perception may partially be due to differences in age distributions between the research circles. The participants in Germany were all >75 years old. In contrast, the participants in Italy, were considerably younger; most of them were <55 years of age. Overall, these types of results are very important to keep in mind for the development of ICT tools that target broad communities of potential users, reminding us of the explicit heterogeneity among older people and people with disabilities.

There were yet other country-specific particularities revealed, such as the type of additional information that was considered important for the new ICT tool to provide: contact information for housing adaptations, costs for barrier removal and housing adaptations, information about which types of personal assistive devices could be useful and where and how to acquire them, information about the availability of accessible transportations and accommodations, educational information on accessibility issues, illustrative pictures of environmental barriers, architectural blueprints of houses, tips on how to arrange furniture, *etc.* Given the marked differences regarding such policies and practices across Europe, the development of an ICT tool with capacity to provide valid and updated information is a major challenge. The results of the present study raise our awareness of major culturally dependent differences and highlight that country-specific tailoring will most likely be necessary for such a tool to reach its full potential.

Overall, there are many accounts of low up-take of ICT products because their development has been technology- or producer-led rather than user-led [32], and older people often experience multiple problems in accessing and using ICT tools [33]. In the present study, we decided to use the HE as the methodological platform because it is one of the few research-based instruments targeting accessibility and it has been empirically tested in various national contexts [15]. Valid problem identification is a necessary condition for the planning of effective action [34], not the least to be able to provide appropriate advice to older people. Accordingly, with a high ambition regarding a scientifically sound base for the ICT tool under development and prerequisites for scaling up this social innovation, there are of course a wide range of other housing aspects that need consideration (see e.g., [8]) including meaning of home, environmental facilitators, and more.

In line with the ambitions of the innovAge project, we applied an approach that engaged potential users, which will address other important product characteristics beyond those identified by the researchers and producers. User relevance is the key to success of intervention and innovation efforts [35], and thus the development of social innovations must be effectuated in close collaboration with the intended user groups. As demonstrated by the results generated in our research circles, the engagement in a collaborative process that occurs among researchers, users and stakeholders [34] is promising as an avenue for knowledge transfer and joint development of new knowledge.

Reflecting upon the method chosen for this project, to the best of our knowledge, this is the first study where the research circle methodology [21,22] has been applied in a cross-national research project. When working in cross-national multi-language research projects it is important, already in the planning phase of data collection, to write up careful guidelines for how to handle challenges in a structured and predefined way [36]. Guidelines should be followed up in joint workshops where national teams come to terms about the data collection and analysis procedure. This step should not be under estimated.

As to alternative methods, the focus group methodology [37] is often used in this kind of research. Similar to research circles, in focus groups, people with similar interests or objectives, discuss particular issues, moderated by researchers. However, there are marked differences as the focus group methodology was designed to give the researcher the opportunity to understand the way people view their own reality. The moderator learns from the participants [37]. In contrast, in the research circle methodology researchers and participants collectively develop new knowledge and learn together [21,22]. That is, the explicit participatory design focus through the use of the research circle methodology made it possible to collaborate with the intended users through the process, creating a forum where new insights were jointly reached. Thus, using research circles produced the form and extent of data that we aimed to achieve, and statements by the participants speak to the positive impact on the creativeness and goal orientation of the process. The choice of method also has an impact on the presentation of findings [23]. Since the aim of this study was to analyse gathered experiences about needs and expectations regarding housing issues in four different countries in Europe, the method chosen had a participatory focus which did not aim for deeper analyses of feelings and emotional behavior. Moreover, involving older people living in diverse countries representing Eastern, Central, Southern, and Northern Europe in a study on ICT development comes with some challenges. For example different housing legislation, different housing options for older people and not the least different research traditions in involving users and stakeholders in the research process.

Another methodological issue worth reflecting upon is that all too often when developing and implementing social innovations such as ICT tools geared towards societal challenges, there is a distinct absence of the involvement of potential users in the process. By involving users in their capacity as consumers with the right to express their opinions [38], they become interesting and useful partners for research leading to efficient implementation and use of resources. In one of the few peer-reviewed studies found that involved users in research on the development of an ICT tool, people with dementia, their significant others and professionals were involved in the process to design a user-friendly videophone [39]. The study revealed different perspectives of the need and design of an easy-to-use videophone. Even so, there were more similarities than differences in the participants’ viewpoints. According to Fudge *et al.* [40], user involvement leads to more relevant research and findings that are more likely to be implemented. In addition, such research might empower the public, which in our case is the growing population of senior citizens.

## 5. Conclusions

Using the research circle methodology to involve older people, their organizations, professionals and experts from the start of a project is promising for the development of social innovations in cross-national research. With the results of the present study, common concerns as well as cross-national differences regarding user priorities and housing options were identified, which highlights the considerable challenges in developing a new ICT tool for widespread use across Europe. Notwithstanding, our findings demonstrate that a user-friendly ICT-tool that makes it possible for older people and people with disabilities to identify accessible housing based on individual needs and expectations is highly relevant and would be appreciated by a wide range of users. Looking ahead, such a tool might contribute to a reduction of the needs for individual housing adaptations and relocation caused by accessibility problems. Ultimately, since accessible housing and neighborhoods support daily activity, mobility and societal participation, a user-friendly ICT tool empowering older people and people with disabilities to be more active consumers regarding housing provision might even have positive effects on healthy life expectancy developments.
